# Bruce Springsteen’s women fans and the deep significance of creative works: meaning, identity, support, and relationships

**DOI:** 10.3389/fpsyg.2026.1751742

**Published:** 2026-04-13

**Authors:** Lorraine Mangione, Donna Luff

**Affiliations:** 1 Antioch University New England, Keene, NH, United States; 2 Boston Children’s Hospital/Harvard Medical School, Boston, MA, United States

**Keywords:** arts, Bruce Springsteen, fandom, meaning-in-life, women fans

## Abstract

Works of art can be extremely meaningful and important to people, and perhaps even more so when people consider themselves fans of a particular artist. In this paper, some of the thoughts and feelings of women fans of Bruce Springsteen are described and discussed, based on two large surveys of the fans. Some of the themes that emerged from the first survey are the relationship women have with Springsteen and his work, the meaning his work has for them, the ways in which his work has been helpful to them, the community of fans that exists, and the loss of longtime band member Clarence Clemons. Themes from the second survey include Springsteen’s legacy, and the ongoing community, especially through the Covid pandemic. The importance of art, in this case, music, for people is emphasized, and suggestions for how psychology can become more involved in the intersection of art and personal growth, identity, well-being, and relationships are offered.

## Introduction

How and why did it happen that two scholars, both academically oriented and both fans of Bruce Springsteen, would come to undertake a large survey research project on Springsteen’s women fans? In our book, *Mary Climbs In: The Journeys of Bruce Springsteen’s Women Fans* (Mangione and Luff, 2023), which discusses this research in depth, the introduction opens with a description similar to this one to begin to tell the story as we lay the foundation for the research. Two women, a clinical psychologist who teaches graduate students and a medical education specialist with a sociology doctorate, sit together in the sociologist’s home to watch a video of a fan’s love for Bruce Springsteen, “*Springsteen & I.”* They had never met before, but were brought together due to their fandom and academic interests when they both volunteered to write a review of this video for the Springsteen online journal, BOSS. They also both felt the enthusiasm about the Springsteen symposia that were happening in New Jersey every few years. A couple of hours later, they were on their way to becoming researchers and writers together, focusing on Springsteen’s women fans, launching surveys, publishing articles and a book, and sharing their work with Springsteen fans and the academic world, and they wrote the review (Luff and Mangione, 2014).

Inspired by Cavicchi’s (1998) book, *Tramps Like Us*, in which he explores Springsteen’s fans through interviews, they heard what he noted about the experience of women fans needing more attention. They also felt that the stereotypes of women fans of male rock stars, focusing on attraction, were not what they had experienced in their years of following Springsteen and his work. Cavicchi’s use of the religious term “conversion,” as it relates to becoming a Springsteen fan, resonated with them and those fans they knew, that becoming a fan could be a major turning point in one’s life and one’s psyche. Coles (2003), who also wrote compellingly about the meaning of Springsteen’s work to fans, was another early influence.

At the time there were several books that included individual fans’ ideas and experiences, such as edited volumes by Womack et al. (2012) and Izzo (2011), as well as work that sought to base itself in both the author’s experience and that of other fans through messaging boards, personal contacts, concerts, etc., with an explicitly spiritual theme to it (Randall, 2011). This has continued with newer books about the experience of Springsteen’s work by authors in edited volumes (Cohen and Sawyers, 2019; Wolff, 2018) or Massaro’s (2021) personal narrative on Springsteen and masculinity. Given this strong personal and academic foundation, our hope was to do something more generally representative, more situated in empirical research, casting a wider net, hearing and sharing the voices of a range of women who felt Springsteen and his work were important to them and their lives. Several years after our first survey, Häkkänen-Nyholm (2021) from Finland, noting that psychology had not included much work on fandom, also did survey research with 600 Springsteen fans throughout the world and showed some overlap of themes with our work. She noted that “females scored significantly higher on regulating arousal and mood with Springsteen’s music than males” (p. 699), and “the findings suggest that for Springsteen’s fans, the primary function of listening to his music is to achieve self-awareness. This is in contrast with findings in music listening in general, which has suggested that regulating mood is the most important function of music listening” (p. 700). While these two aspects, self-awareness and regulating mood, are different and may even be considered antithetical, they both seem important to the experience. Within psychology, Kalichman and Smyth (2023) did a text analysis on Springsteen and authenticity in his lyrics on 18 studio albums, an important concept since a musician’s authenticity can feel very important to fans. Authenticity “is defined as conveying a sense of truthfulness and being forthright in self-presentation” (p. 581). While the results are very nuanced and layered, and the comparisons they make with other musicians are complicated, Springsteen’s authenticity does continue throughout the decades, with some changes over time.

Fandom is an area that has its own area of study, typically called Fan Studies, Cultural Studies, or as part of an American Studies program, which contributed to our thinking for our study, and which continues to grow and evolve. Early work was brought together in the 1992 book edited by Lewis, *The Adoring Audience*, which included chapters on many facets of fandom, with one that delved into negative aspects of fandom and sought to separate more cultish or pathological aspects and views of fandom from healthier and more mainstream experiences (Lewis, 1992; Jenson, 1992). Current writings on sports fans also look at more problematic aspects of fanaticism (Wojtowicz et al., 2026). Part of the motivation for this current research was the desire to examine what we knew was also out there: more positive or complicated reasons for fandom. Duffet’s (2013) far-reaching writings on fandom that really broadened the concept helped in laying the groundwork for us in thinking about fandom as an area of research. It also noted the complexity of the issue of cults and even the word itself. Duffet brought fandom into the mainstream for academics, and maybe for fans and society’s views of fans as well, a perspective that our work definitely holds. Springsteen’s fans are mentioned throughout Duffet’s work, particularly with regard to the Cavicchi book that so influenced this current research. Stever and Tukachinsky Forster (2025) offer a comprehensive review of Fan Studies and its intersection with psychology, particularly social psychology, with an emphasis on social identity theory, and with the area of parasocial relationships. Identity and relationships are central to the Springsteen fans’ research. They also address the issue of whether women fans, particularly older women fans, are sometimes described in negative ways, another viewpoint with which our work resonates. Types of fans that are studied include sports fans (Hirt and Clarkson, 2011), an area that can sometimes feel similar to fans of music performers and seems to have only gotten more pronounced as the years go by. The concept of “parasocial relationships” or the feeling that one has a relationship with a celebrity has become more popular in fan studies (Stever and Tukachinsky Forster, 2025; Hartmann, 2016; Weimer et al., 2022). Recently, a student in my department successfully finished her dissertation that involved a complex look at fans of the Jonas Brothers (D'Anna, 2025), and another student and a faculty member have brought psychology research to the fandom of Taylor Swift (Hardock et al., 2025). Swift’s rise to fame and her relationship with her fans is a growing area of interest, perhaps best described in a recent book on Swift by McCann et al. (2025) that goes into great detail about her career, current society, and fans It can also serve as a reminder to those who study fandom that the way fandom is experienced and expressed is dependent on the societal, cultural, and technological events and possibilities at any given time.

Writers in Fan Studies, who are from a variety of disciplines, have also considered Springsteen’s work. Through her own very detailed critical reading and feminist analysis of the lyrics of four of his studio albums, Moss (2011) explores women’s roles in Springsteen’s work and seems to see their roles as complicated and multi-layered but as secondary to men’s more central roles. Baxter-Moore (2016) examines why Springsteen fans attend concerts, even multiple concerts in one tour, using his own experience as well as responses through fan websites, with the focus more on the aspects of the concert rather than the fan’s internal experience. Emotional responses to one particular band member’s appearance and the importance of community were, however, noted. Yates (2015) conducted a survey that he described as a “pilot investigation” (p. 254) to see how Springsteen fans use the internet to engage in a parasocial relationship with Springsteen, yet found that the more important use of the internet was to connect with other fans. “The findings do indicate that Springsteen fans utilize the Internet to develop social relationships with one another and to form a worldwide community that centers on Springsteen” (p. 261). He also notes some differences between male and female fans, such that “females, not surprisingly, find Springsteen attractive, tend to seek out other stories about Bruce in traditional print media, and have a greater desire to meet Springsteen in person than males” (p. 266).

We also wanted to bring our disciplines of clinical psychology and sociology into the conversation, as they had a lot to offer. While sociology does include work in popular culture and the arts, the arts and their effects on people have not typically been emphasized in psychology, especially not in clinical psychology, although that seems to be changing. In fact, the American Psychological Association’s publication, the *Monitor on Psychology*, recently included an article looking at “Art and the Mind” (Weir, 2025), which did emphasize the importance of art in people’s experience, including a variety of researchers and projects, with a special focus on art and its effects on the brain. Researcher Susan Magsamen is quoted as saying that “Artists, philosophers, and children have always known the power of the arts. It’s innate and intuitive, yet we have marginalized the way we have thought about the arts as a form of entertainment…. In fact, the science shows we are neurologically wired for these kinds of experiences, and they contribute to our health and well-being” (Weir, p. 56). This fits with what we observed in our research. A systematic review of transformative experiences and the arts by Pizzolante et al. (2024), mentioned in this article, noted the connection between the arts and transformation and suggested it was something for more research and for consideration in therapy. Hopefully, this Springsteen research will add to that consideration.

In this article, we focus on how creative products and performances can have a transformative effect on fans’ lives, sense of self, sense of wellbeing, meaning in life, and identity. This can be seen in the intersection of the creative product with fans’ thoughts, feelings, life experiences, and reflections. Creativity and creative products/performances can bring deep support and encouragement to fans, can help with seeing oneself and one’s experiences in a new light, as well as enhancing relationships and building a community. We also hope to engage the world of psychology with what can be meaningful from the arts and through fandom regarding all the areas noted above: the transformative effect on people’s lives.

## The two surveys

At the time of these surveys, 2014 and 2021, a much-visited and much-loved fan site called Backstreets.com existed, which was welcoming of hosting the surveys. This allowed us to reach Springsteen fans more directly, yet even so, we were surprised by the number and intensity of responses.

These are the major questions from our first survey, really trying to get to the depth and meaning of their fandom. We wanted the questions to be open-ended so the respondents would feel free to think about and answer them in ways that felt most fitting for them. We hoped to hear their stories, and each question prompted such responses:

- When and how did their fandom start?
- What is it that women fans get from Springsteen’s music?
- Why is he important to them, and in what ways?
- How do they feel about his writing and perspectives on women?
- Do they feel they have a relationship with Springsteen?
- Has Springsteen’s music ever helped them in their life?

While we were hoping for 50–100 responses, we shut the survey down after about a week as we had 1,152 responses with 908 that completed the demographics from 44 states in the United States and Washington, DC, and 24 other countries, with an age range of 15–88 years old. The responses were often long and rich in detail and emotion.

We decided to do a second survey in 2021, given the pandemic and new albums and activities by Springsteen in the intervening years. These are the questions from our second survey:

- In the past few years, Bruce Springsteen has released a number of new works and performances. Please let us know which of these you have heard/seen and how important they were to you/what you thought of them.
- Can you describe in three words or short phrases what you think Springsteen’s legacy will be?
- In what ways, if any, has your experience as a Springsteen fan changed in the last few years?

There were 742 responses, with 434 completed demographics, from the United States and 22 other countries, with a range of ages from 15 to over 75. Our book, *Mary Climbs In: The Journeys of Bruce Springsteen’s Women Fans* (2023), offers more details about the surveys and the data. From the first survey, a book chapter (Mangione and Luff, 2018) and an article (Mangione and Luff, 2019) have also been published. These surveys are the foundation for this article, and all of the quotations included here are ones not published previously.

## Themes from the first survey: relationships, help, and Hope on the journey

When the art form one is investigating is a performance, with the artist in the role of, say, a musician, an actor, or a dancer, the relationship with the art becomes enmeshed in the relationship with the artist. One is not looking at and experiencing a painting by an artist one does not know or see; rather, the artist is “delivering” the art, so to speak, and embodies the art. It is hard to separate the artist from the art, as the experience is more holistic. Therefore, in studying women’s relationship to Springsteen’s music, we also needed to seek to understand their relationship to Springsteen himself. The following themes incorporate Springsteen’s work, along with himself as the one who creates and presents that work. We found three major relational themes that embody the significance of the work and the artist described below. These relational themes are significant because they provide the foundation for Springsteen’s work to have such an influence on his women fans in the areas described above, such as their lives, sense of self, sense of wellbeing, meaning in life, identity, support and encouragement, relationships, and community.

### Family/friend

We asked about their relationship with Springsteen, what Springsteen means to them, and a family member or friend was often noted. Some said it very simply: “He is like a friend that you love and you want to do well. And he always does.” For others, it evolved over time: “I thought the skinny kid with long hair was sexy. When he cut it and started working out, I was more attracted to the music and thought of him more like a brother than a potential lover.” Others fleshed out their experience a bit more: “Bruce’s integrity in living a normal life (taking Elvis’s example and turning it 180 degrees) to give his kids a healthy upbringing out of the spotlight and away from the craziness of show business. He projects an aura of approachability—I think of him like a brother (he does have my brother’s nose) on whom I can depend.”

This fan described the feelings behind seeing him as her best friend: “Yes. I feel that I do have a relationship with Springsteen as a fan. He does not know that he’s my best friend, but he is. His music/lyrics speak to me and have provided me with so much comfort and happiness over the decades. He could charge much more money for his concerts but tries to keep things reasonable so that fans like me, a single parent school teacher without a lot of money, can still go to see several shows each tour. He puts on shows that are much longer than most artists do and gives 100% of himself to his audience at every show.”

Having some sense of a relationship with an artist can be profound and can be described in different ways. Truly feeling that the artist could be your best friend or brother can both emerge from and add to the impact of the artistic creations, laying the foundation for making the art more meaningful and important. The whole experience can feel more personal and, therefore, probably more impactful.

### Teacher/mentor/spiritual guide

Some women went on to describe their relationship with Springsteen as a teacher, mentor, or spiritual guide, using those words either very simply or elaborating upon them, and sometimes describing themselves as a student. This description speaks to the layers of the relationship, and perhaps the complexity too: “He is like a father, teacher, brother and lover to me. His music guides me through life.” There is a real sense that Springsteen and his work have given them something important for their lives and learning, something that defies simple categorization. This response differentiates kinds of relationships, but ties in the spiritual piece and how it affects her: “Well obviously not a ‘hey come have a beer with me for happy hour’ relationship. To me, it’s more of a spiritual awakening by coming to terms of certain life events, brought forth by hearing those lyrics that reach deep down into your inner most self. But I think it would be fun to go grab a beer with him if the opportunity presented itself!” Probably many fans would relish the beer with him and also appreciate and grow from the lyrics that go deep into oneself.

When asked if Springsteen’s work had helped them, participants felt that it helped them in different ways. “It’s more like a spiritual guideline.” “His music helps me every day. It lifts me when I need it, it keeps me company when I am lonely, it guides me when I am unsure of something.” “Without his music I think I would not make it so far. His songs comfort me.” There is something real and inspiring in his work, something that fans take seriously, that affects their lives and who they are. From comfort to inspiration to guidance, the music is there for them. Again, we see how the relationship they feel they have with him adds to the impact of his work on them, acknowledging how important the work is in their lives and their functioning. He and his work represent something outside of themselves, yet clearly involved with who they are.

### Therapy/healing/hope

Springsteen as a therapist or his work as therapy were both named in the responses, and some described that in more detail. “I jokingly call him “The Preacher Man” or “The Therapist” because of his songs and the release that occurs in all of his shows. His shows combine the stories of people who should matter but do not, giving you pause to think about the bigger and smaller pictures in your life, with a silly rock-and-roll sing-along dance-along, which everybody needs now and again to stay sane.” The concerts are part of the healing, and both the fun and the thinking about life almost seem to join together.

This woman also brings in the idea of Springsteen as a therapist, even though she has her own therapist in her life. “As I was driving to work the other day I heard a song on E Street Radio that really touched me. I believe it was an out-take from Nebraska or that era, so I do not know the name of the song, but it was such a heartfelt song about two lonely people. I find Springsteen’s ability to put his finger on such raw emotion and feeling for the individual people in his songs a gift. I joke with my therapist that he was my ‘first’ therapist because I could relate to so many of the things he sang about. Bruce has also been a source of fundamental goodness and fun in my life as well.” Being able to relate to Springsteen’s work, just as one would expect to relate to one’s therapist, seems to lay the groundwork for this “therapy.”

Depression is a deep and layered experience, and a person can undergo a lot of challenges in trying to get better. This woman’s journey speaks of struggle and complexity. “The depression had been crippling, devastating—what was it that finally defeated the cynicism and isolation, the desperate dark hopelessness that had left me unable to function on even the simplest level? How was I now stepping into a new strength and purpose? What had made me better? I listed a number of things: Exercise made me better. Work made me better. Therapy made me better…. All of those were true. But I missed one that day, one that has also been essential in keeping me moving forward…. So let me say it now. Bruce Springsteen made me better. I became a fan in those days of deep darkness, when I encountered this person who could go darker and deeper, who did not just play at putting a toe over the abyss, but leaped straight in. Here was someone who had no time for self-deception or pretty illusions, someone who wasn’t going to waste time telling lies. And yet, there this person was, so utterly intoxicated by a sense of possibility, by the wonder and beauty and grace of life. Life is a mystery, he was shouting, it’s a ride you cannot control – just stomp on the accelerator and stick your head out the window!”

This person certainly found the depth of meaning she needed as well as the exhilaration to keep moving. The accelerator image, coming after her description of the despair, really shouts out the hope and possibility of moving forward in one’s life. Being willing and able to see a rock musician as a “therapist” for oneself speaks to the power of that artist’s creative work and to one’s openness to letting it affect one’s mood, life, and wellbeing. It gives one a companion for putting a toe over the abyss, an abyss that many people encounter in their lives.

Yet for many, hope is something that can often be hard to find when one is going through difficult times, and it can feel that there is no hope left, given one’s situation. However, these women found hope in Springsteen’s work. “His words have traveled with me. Through leaving home, falling in love, lust, heartache and above all hope!” The word “redemption” speaks to something powerful in these thoughts from a participant. “For a favorite live song, it has to be Land of Hopes and Dreams from the Live from New York album. I’ve seen him sing that song live, and that version never fails to give me chills. It is so full of redemption and second chances, which are a constant theme in Springsteen’s music.” The ability to pair hope with more negative experiences and emotions seems to resonate with this respondent and many of the women. Hope is a very necessary ingredient in people’s lives when going through difficult times, and creative works can have the power to instill or increase hope. Without the hope from Springsteen’s work, the abyss could seem much more threatening.

### Community

Learning, growing, relationships, getting help, holding on, and finding hope—all of this continues for some within the community of fans. “Bruce is one of us. Connected. Passionate. Shows who he is on stage. I learned integrity and how to take care of yourself during his disagreement with Sony. Family relations. Helped me to build a Springsteen family around the world. People ask me what his concerts are like and I say it is like being at the cottage on the lake and your best friends are there with the greatest band on earth.” The cottage on the lake with friends image evokes a real community, a coming together, and the bonds between people. We will address the community again in talking about the second survey since it clearly showed up in both surveys and can be so powerful and needed in people’s lives. Fandom can have a very communal aspect to it, which adds to the power of the creative experience, the living of it with others who also value the experience.

## Themes from the second survey: legacy, celebration, and community

### Legacy: how will Springsteen be remembered?

We asked this question, with which some people complied, but others thought it was not helpful in trying to understand his legacy: Can you describe in three words or short phrases what you think Springsteen’s legacy will be?

We appreciated this very forthright objection. “No I cannot, that’s an absurd question. His legacy already is and will continue to be huge and it demeans it to try and shorten or condense it to a few words.” Another let us know that she did not really resonate with some of his recent work, especially his Broadway shows, and was feeling the losses due to COVID. “I felt like I could see him being very conscious of creating his legacy and putting his energies into all the wrong things (Broadway). I listened to him for comfort a lot during Covid, the Darkness-River-Nebraska era that is my favorite. I miss Springsteen concerts and it makes me feel so old thinking of the lost years due to Broadway and Covid.” Even these kinds of responses speak to the importance of creativity and creative products for fans. They also speak to fans’ ability to take a step back and assess the situation.

Yet others did embrace this huge question and considered what will be his legacy as we see here:

- His great musical legacy and his long exciting live shows.
- Steadfast faith in life, goodness and God Tenacity in all the unexpected roads of life Gifted in the depths of story telling which often are not stories but instead reality.
- He gave voice to the oppressed, the silenced, the damaged, but mostly, the hopeless. Because nothing says It Will Be Better than listening to Thunder Road.
- Patriot, poet, and friend of the powerless.
- Live music and powerful lyrics. Storytelling.
- The Road - Freedom Sensitivity to subject matter The E Street Band.
- Best rock & roll performer of our time, giving it all every show.

One woman had a very different view of the Broadway show than the one mentioned above. “My tix for Broadway were 10 rows from the stage. When I walked in the theater and was escorted to my seat I burst into tears. The usher looked at me and said- “this is a really special show.” He got it. Which told me he saw this same response hundreds of times before. What a legacy to have.”

Springsteen’s legacy may indeed include all of the above, held together by the thread of creativity, as the women seemed to express the essentials of his work in their comments. His social justice aspects and his giving voice to others stood together with his compelling music and enlivening performances. While these responses were not specifically addressed to individual transformation from his work, the power to change and move lives forward, and to speak to layers of societal issues, underlie these responses.

### Celebration and community

Community was mentioned briefly many times in the legacy question, giving the sense that the way community has developed around Springsteen, and his relationship to that community, including honoring and valuing it, will be part of his legacy. Some of the respondents gave greater detail in terms of what the community has meant, such as this one who started traveling partly because of the community. “I’ve become a much bigger fan since I found the online fan community. I’ve made many friends this way, most online, but some in the real world. I began traveling to shows (which I very much enjoy), something I’d never imagined doing previously.” Springsteen’s work, and especially the concerts, have opened up a whole new world for some of his fans. “I have made amazing friends in the Springsteen community who I would never have met.” “There is a strong community of fans that is important to the “Springsteen Experience” as well. I have made friends all over the country who are very like-minded - bruce brings people together and my life is enriched by these friends and the light they bring to my life.” The connection and warmth feel palpable.

Finally, this person says it very simply but with heartfelt meaning. In a world that can so often feel isolating, what a gift this type of community can be. “I have always felt like I am part of a beautiful, friendly worldwide community.” With regard to the worldwide community, both researchers have had the opportunity to experience that excitement and coming together firsthand, in England and in Italy. In a world that can sometimes seem very isolating, the development of a community based on a performer’s creative work can be a powerful experience and can change lives.

## Mourning and memorializing the loss of Clarence Clemons

Clarence Clemons filled any concert venue with the intensity and passion of his saxophone, his very strong presence, and the joyful and powerful interaction between him and Springsteen. In June of 2011, Clarence passed away, leaving a huge empty space for many fans and for the band. The music, either when listening on one’s own or especially at shows that followed his death and included a tribute to Clarence, inspired tears and sadness, but also helped to memorialize and keep Clarence Clemons and his music alive for the band and the fans. Two songs in particular, Jungleland and Tenth Avenue Freeze Out, spoke to his presence and his absence.

The sadness is there, sometimes described as sobbing or tearing up, yet there is a sense of memorializing that keeps Clarence Clemons present and in fans’ hearts. “The last two shows I’ve been to were after Clarence died, so the songs in which he originally had a large part, like 10th Avenue Freeze-Out, are pretty sad, but also amazing in that the whole crowd comes together to celebrate Clarence.” Clarence’s nephew, Jake Clemons, now plays saxophone in the band, and several women commented on that. “Bruce played meeting across the river and then Jungleland in NJ 2013. Brought back many memories of how well it sounded when they did it with Clarence. To have family (Jake) take his Uncle’s place really cements what Bruce is all about.” This comment about Springsteen and family feels fitting. Another band member, who played the accordion and organ, Danny Federici, also has passed away. “Now that Clarence and Danny have passed, Sandy and Jungleland makes me feel sad as they are gone, but then I remember their music is still living.” Yes, the music is still there.

The meaning of losses cannot be overestimated. “Tenth Avenue used to make me happy but when I saw it used as a tribute to Clarence it made me cry and I felt it really put into words how much Clarence meant to Bruce.” It would be hard to miss the meaning that this loss had for Springsteen. The loss of Clarence dovetailed for this woman with another loss in her life. “The only time I remember crying was at a concert in 2012. The Clarence tribute during Tenth Avenue Freeze Out. I had just lost a friend to cancer, and the tears came rolling.” Loss and grief are ubiquitous and can connect to other such experiences.

Clarence’s passing intertwined with a woman’s relationship with her father, and she voices the gratitude she feels toward Clarence, also called the Big Man. “Jungleland will always be near and dear to my heart because of the relationship I have with my father. All Springsteen music is good to listen to with the windows down while driving, but when I’m driving and listening to Jungleland, something else happens. I think about the Big Man every single time I listen to this song. In Philadelphia 2012, when the band did the dedication during Tenth Avenue, I saw a tear run down my father’s cheek. I get my passion for Springsteen’s music from my father, and the connection he has with Clarence’s music was also passed to me. I have an experience when I hear that sax. It moves me. I point to the sky and always say, “Thank you Big Man,” when I’m driving.” Clarence is still there, in some form or another, for this woman and for several others.

That certainly comes close to feeling like a prayer, and, once again, reminds us of how vital fandom, creativity, artists, and relationships are in people’s lives. In a culture that can sometimes minimize loss and grief and urges people to “get over it,” this resonates with the loss of Clarence Clemons, which can be seen as a real gift to fans and invokes Clemons’ great creativity and the strong sense of a fan community. It may be validating the experiences they have had with losses of their own friends and families.

## In the end: the importance of creativity and fandom

Those whose intense engagement with Springsteen’s work described above has shown the value, importance, and depth of engaging with an artist’s creative productions, and how significant that work can be to one’s life and oneself. In this final section, a summary of the research presented, some conclusions along with other literature, limitations, and suggestions about the interface of creativity and one’s experience of it are offered. This includes ways in which the field is moving forward in the study of creativity and its impact, and how psychologists can include that experience in other ways in their work.

### Layers of fan experience

Numerous women fans of Bruce Springsteen from various states in the United States and several other countries were surveyed, and the results show the value and importance of his work for these fans as they engaged with his music and performance. Many of them saw him and his work as having real relevance to their lives, their personal growth, the issues they faced, and who they are as people. Seeing Springsteen as a family member or friend, teacher or guide, or therapist or helper were themes in the responses that conveyed the deep meaning he and his work had for them. Hope is something many people need, and being a Springsteen fan can add hope for some. The power of the community that comes together as fans, a feeling of camaraderie and belonging with other fans, enhanced their relationship with Springsteen and his work, making their fandom even more compelling for them. Responses that brought in the loss of Clarence Clemons, an iconic and beloved member of the E Street Band, exemplify the sense of connection and significance felt by fans. Noting Springsteen’s connection to the marginalized and the powerless, the social justice aspects of his work spoke to some fans as they thought about legacy.

Community and hope are two aspects of women’s responses that stand out, particularly given our current world in which the Surgeon General of the United States has spoken out about the lack of community and the prevalence of loneliness and isolation (Murthy, 2025). The loss of hope is typically a concern with many kinds of mental health problems, particularly depression. In group therapy, instillation of hope is considered one of the core therapeutic factors (Yalom and Leszcz, 2020), given that people often enter a group feeling little hope. Hope theory, developed by C. R. Snyder, reconfigured hope and emphasized that it is active, realistic, and purposeful, moving one forward (Snyder, 1994; Snyder, 2000; Snyder and Lopez, 2009), and research in this area of hope continues and looks to expand how hope is studied and framed (Colla et al., 2022). Community and hope, both of which have been experienced by Springsteen fans, seem increasingly valuable and necessary, and for these women, their relationships to Springsteen, his work, and other fans are one way to form and develop these. As the current author wrote in conjunction with a co-author long before this research, “We argue that for Springsteen, healing and transformation lie within relationships, even complex and difficult ones” (Mangione and Keady, 2007, p. 180). This still feels relevant almost 20 years later.

Creativity and artistry have the power to enhance and even transform the lives of those who participate with the artistic products and performance in the areas noted throughout this article: fans’ lives, sense of self, sense of wellbeing, meaning in life, identity, support and encouragement, relationships, and community.

### Limitations

Limitations of this research include that it was not comparative in that we do not know what men who are fans might have said, and we cannot state what is specifically relevant only to women fans. With such a large data set, it is also important to remember that varying viewpoints exist, despite the emerging themes. We can also not verify that all the respondents identified as women, given that it was a survey.

### Research and the arts: moving forward

Engaging with creative products, such as music, can have a meaningful impact on people, and perhaps especially when people consider themselves fans of a particular artist. Fandom can encompass significant depths, nuance, power, and complexity. The capacity of creativity to transform lives and bring people together should not be underestimated. Researchers in psychology do seem to be focusing more on art’s impact on people (Weir, 2025), highlighting how important it can be in people’s lives and development. A summary of that article suggests that “Esthetic experience may promote positive outcomes such as empathy, social connection, and cross-cultural understanding” (Weir, 2025, p. 56). While that may seem like a large expectation, our Springsteen research suggests that we are starting to see some of this, and psychology can hopefully better utilize art and creativity. Emotional valence was seen as important for the experience of music in a study of people’s responses to various art forms, and emotional arousal has often been cited in music (Mehl et al., 2025), which fits with our current research noting a deep connection between affect and music. However, it is interesting to note, given that Springsteen’s work can be very poetic, that in this study (Mehl et al., 2025), vividness of evoked imagery was especially important for poetry’s appeal, so a mixture of emotional valence and vividness might be seen in Springsteen’s work and other music that resonates with people.

A current review of a process called esthetic cognitivism, which refers to the idea that “art can promote new knowledge and understanding” (Christensen et al., 2025), while focused on visual arts, still feels relevant to the experience of art described here. Their general question, as they review ideas and research in favor of and opposed to esthetic cognitivism, resonates with our work: “Do people gain new knowledge or understanding from artworks?” (Christensen et al., 2025). While the article concludes with the difficulty of empirically finding answers to their many questions of esthetic cognitivism, the present work on Springsteen fans resonates with a positive view of gaining knowledge and understanding through the arts. A recent empirical dissertation “examined the ability of different kinds of music interventions to reduce negative emotions and increase positive emotions after a stressful event” (Fountain, 2023), looking at emotion regulation through music, an area that has been researched and used in certain types of clinical practice for many years. The author concludes with suggestions for clinicians in terms of aiding clients in using a self-selected playlist of music for help with negative emotions. Wilt et al. (2025) discuss the learning and understanding that can be gained through creating art or interacting with art in a research project looking at the types of knowledge and understanding that can be potentially gained, with a foundation in esthetic cognitivism, in conjunction with McAdams’ (2011) idea of the narrative self, a concept that has been discussed in relation to our research. Wilt et al.’s (2025) aim is to “bring together esthetic cognitivism’s concern around knowledge gained from art with narrative identity’s focus on stories dealing that have existential themes” (p. 1). They used structured interviews around creating art and interacting with art. Their findings “suggest that engaging with art can lead to deeper existential understandings within the context of narrative identity and meaning-making” (p. 15). They also “highlight the potential value of art as a tool for existential exploration” (p. 16).

While all of these studies and reviews cited here offer limitations to their work and cite the complexity of the issues and the research, it is clear that the power of the arts, and in this case, music, has some foundation in research. The research with women fans cited throughout this article, although of a different approach and genre than these articles, lends another kind of “evidence” and substance to this research on the significance of art and creativity.

### Integrating the arts into psychology

Psychologists engaged in therapy, assessment, or supervision could consider the impact of art and fandom on clients and ask about it as a way to understand the client’s world, values, resources, and possible online or in-person communities. Those thoughts and feelings can be incorporated into the work they are doing together. Even though sometimes described as a “therapist,” Springsteen is not actually a therapist, but his work and that of other artists can be part of therapy. It can also resonate with a sense of spirituality and/or religion, a much-overlooked diversity in clinical practice, but noted in this research and Randall’s (2011) work on Springsteen, fans, and spirituality. In a chapter looking at a research study on wellbeing and fandom with youth in India, the author (Poyil, 2025) suggested that “Mental health professionals might consider incorporating fandom-related activities in treatment plans to promote well-being.” It seems like the field of psychology is starting to recognize the importance of fandom for some people, as well as the arts in general, and how both can promote personal growth and connection. Certainly, ideas of fandom as deviant or pathological (Jenson, 1992) or the fanaticism of some fandom noted in the introduction (Wojtowicz et al., 2026), while perhaps important for understanding certain fans, need to allow for so much else that has been researched in the fan world since then and shows a much more positive possibility for fandom’s meaning and purpose.

The arts can also be a way to engage a reluctant client by seeking to understand an important part of their world. This could be especially necessary cross-generationally when an adult psychotherapist needs help understanding a younger client’s relationship to a certain artist or performer and can learn a lot by listening to the experience of the client around music or books, for example, or a young student in training has little idea about who might be considered meaningful to an older client. Faculty can help students find compelling research projects involving art and artists. Heads of organizations can use fandom to find common ground or start conversations among employees. Loss and grief often evoke connections to music that may have been important for the deceased or may help those who are mourning, something this author includes in workshops and groups on loss and grief, and has been seen in her prior research in this area with Italian American daughters and dads (Dicello and Mangione, 2015).

The arts can and should be more infused throughout our work as psychologists, whether as researchers, clinicians, teachers, supervisors, consultants, administrators, or leaders in organizations or the field. The arts can enhance our humanity, our learning, our growing, and our very being.

## Data Availability

The datasets presented in this article are not readily available because of the possibility of identities being known. Requests to access the datasets should be directed to lmangione@antioch.edu.

## References

[ref1] Baxter-Moore N. (2016). “The ties that bind”: Springsteen fans reflect on the live concert experience. Rock Music Stud. 3, 80–104. doi: 10.1080/19401159.2015.1129831

[ref2] Cavicchi D. (1998). Tramps like us: Music and Meaning among Springsteen fans. New York City: Oxford University Press.

[ref3] Christensen A. P. Cardillo E. R. Chatterjee A. (2025). Can art promote understanding? A review of the psychology and neuroscience of aesthetic cognitivism. Psychol. Aesthet. Creat. Arts 19, 1–13. doi: 10.1037/aca0000541

[ref4] Cohen J. D. Sawyers J. S. (Eds) (2019). Long walk home: Reflections on Bruce Springsteen. New Brunswick, NJ: Rutgers University Press.

[ref5] Coles R. (2003). Bruce Springsteen's America: The People Listening, a poet Singing. New York City: Random House.

[ref6] Colla R. Williams P. Oades L. G. Camacho-Morles J. (2022). “A new hope” for positive psychology: a dynamic systems reconceptualization of hope theory. Front. Psychol. 13:809053. doi: 10.3389/fpsyg.2022.809053, 35282244 PMC8906075

[ref7] D'Anna S. (2025). The Jonas brothers and you: a literature review on the impact of Parasocial relationships. In: Chickerella R. (Chair), “That’s just the way you make me feel”: music fandom and media representation among young adults. Symposium for Division 46. Presented at the American Psychological Association 2025 Conference. Denver, CO.

[ref8] Dicello D. Mangione L. (2015). Daughters, Dads, and the path through Grief: Tales from Italian America. Atascadero, CA; Oakland, CA: Impact Publishers/New Harbinger.

[ref9] Duffet M. (2013). Understanding Fandom: An Introduction to the Study of media fan Culture. New York City: Bloomsbury Academic.

[ref10] Fountain C. (2023). Silver linings playlist: exploring the effectiveness of music as an emotion regulation tool. Electronic Theses and Dissertations. 2366. Available online at: https://digitalcommons.georgiasouthern.edu/etd/2366 (Accessed October 29, 2025).

[ref11] Häkkänen-Nyholm H. (2021). Bruce Springsteen fan behavior and identification. Psychol. Music 49, 691–703. doi: 10.1177/0305735619891774

[ref12] Hardock T. D'anna S. Chickerella R. (2025). The “Gaylor” phenomenon: a content analysis of Reddit users questioning Taylor swift’s sexuality. In Chickerella R. (Chair), “That’s just the way you make me Feel”: Music Fandom and media Representation among young Adults. Symposium for Division 46. Presented at the American Psychological Association 2025 Conference. Denver, CO.

[ref13] Hartmann T. (2016). “Parasocial interaction, parasocial relationships, and well-being,” in The Routledge Handbook of media use and well-Being: International Perspectives on Theory and Research on Positive media Effects, eds. Reinecke L. Oliver M. B. (New York City: Routledge Press), 131–144.

[ref14] Hirt E. R. Clarkson J. J. (2011). “The psychology of fandom: understanding the etiology, motives, and implications of fanship,” in Consumer Behavior Knowledge for Effective Sports and event Marketing, eds. Kahle L. R. Close A. G. (New York City: Routledge/Taylor & Francis Group), 59–85.

[ref15] ed. Izzo D. (2011). Bruce Springsteen and the American Soul: Essays on the Songs and Influence of a Cultural icon. Jefferson, North Carolina: McFarland and Company Incorporated.

[ref16] Jenson J. (1992). “Fandom as pathology: the consequences of characterization,” in The Adoring Audience, ed. Lewis L. (New York City: Routledge).

[ref17] Kalichman S. C. Smyth J. M. (2023). “And you don't like, don't like the way I talk”: authenticity in the language of Bruce Springsteen. Psychol. Aesthet. Creat. Arts 17, 581–589. doi: 10.1037/aca0000402

[ref18] Lewis L. A. (1992). The Adoring Audience. New York City: Routledge.

[ref19] Luff D. Mangione L. (2014). Springsteen & I: “friends since…” movie review in BOSS, on-line journal on Bruce Springsteen’s work. BOSS—The Biannual Online-Journal of Springsteen Studies. 1, 124–128.

[ref20] Mangione L. Keady S. (2007). “Spirit in the night” to “Mary’s place”: loss, death, and the transformative power of relationships. Psychol. Aesthet. Creat. Arts 1, 179–190. doi: 10.1037/1931-3896.1.4.179

[ref21] Mangione L. Luff D. (2018). “Who is Springsteen to His Women Fans?” in Bruce Springsteen and popular music: Essays on Rhetoric, social Consciousness, and Contemporary Culture, ed. Wolff W. I. (New York City: Routledge Press).

[ref22] Mangione L. Luff D. (2019). Women fans’ journeys through darkness. Interdiscip. Lit. Stud. 21, 26–41. doi: 10.5325/intelitestud.21.1.0026

[ref23] Mangione L. Luff D. (2023). Mary Climbs In: The Journeys of Bruce Springsteen’s Women Fans. New York City: Rutgers University Press.

[ref24] Massaro J. (2021). Shades of Springsteen: Politics, love, Sports, and Masculinity. New Brunswick, NJ: Rutgers University Press.

[ref9001] McAdams D. P. (2011). Narrative identity. In Handbook of identity theory and research. Eds. S. J. Schwartz, K. Luyckx and V. L. Vignoles. (Springer Science + Business Media), 99–115. doi: 10.1007/978-1-4419-7988-9_5

[ref25] McCann H. Faichney E. Trelease R. Whatman E. (Eds) (2025). Taylor Swift: Culture, capital, and Critique. New York City: Routledge.

[ref26] Mehl K. Gugliano M. Belfi A. M. (2025). The role of imagery and emotion in the aesthetic appeal of music, poetry, and paintings. Psychol. Aesthet. Creat. Arts 19, 1373–1382. doi: 10.1037/aca0000623

[ref27] Moss P. (2011). Still searching for the promised land: placing women in Bruce Springsteen’s lyrical landscapes. Cult. Geogr. 18, 343–362. doi: 10.1177/1474474011410276

[ref28] Murthy V. H. (2025). My parting prescription for America. Office of the U.S. Surgeon General.

[ref29] Pizzolante D. Pelowski M. Demmer T. R. Bartolotta S. Sarcinella E. D. Gaggioli A. . (2024). Aesthetic experiences and their transformative power: a systematic review. Front. Psychol. 15:1328449. doi: 10.3389/fpsyg.2024.1328449, 39421842 PMC11484404

[ref30] Poyil F. S. (2025). Fandom and wellbeing - exploring the psychological link. Advances in the Psychology of Well-Being. ed. S. Spowart, 69, doi: 10.5772/intechopen.1009519

[ref31] Randall L. K. (2011). Finding grace in the concert hall: Community & Meaning among Springsteen fans. Long Grove, IL: Waveland Press, Inc.

[ref32] Snyder C. R. (1994). The Psychology of hope. New York City: Free Press.

[ref33] Snyder C. R. (2000). Handbook of hope: Theory, Measures, & Applications. New York City: Academic Press.

[ref34] Snyder C. R. Lopez S. J. (Eds) (2009). Oxford Handbook of Positive Psychology. New York City: Oxford University Press.

[ref35] Stever G. S. Tukachinsky Forster R. (2025). “The psychology of fandom and parasocial experience,” in The Oxford Handbook of Media Psychology, eds. Shackleford K. E. Bowman N. D. (Eds). 2nd ed (New York City: Oxford Library of Psychology), 450–467. (2025; online edn, Oxford Academic, 22 July 2025)

[ref36] Weimer C. E. Riles M. J. Tewksbury D. (2022). Artists and attributions: how music platform implementation affects parasocial experiences and support intentions. J. Broadcast. Electron. Media 66, 300–319. doi: 10.1080/08838151.2022.2086550

[ref37] Weir K. (2025). Art and Mind. Monit. Psychol. 56, 54–61.

[ref38] Wilt J. A. Exline J. J. Sherman A. Schlegel R. J. Swartz S. Blanco L. . (2025). Behind every artwork is a story: phenomenological analysis of understandings achieved through engagement with art. Psychol. Aesthet. Creat. Arts. Advance online publication. doi: 10.1037/aca0000817

[ref39] Wojtowicz J. Archer A. Fruh K. (2026). Fans and fanaticism: the vulnerability of devotion and sportswashing as exploitation. Philos. Psychol. 39, 175–197. doi: 10.1080/09515089.2025.2499626

[ref40] ed. Wolff W. I. (2018). Bruce Springsteen and popular music: Essays on Rhetoric, social Consciousness, and Contemporary Culture. New York City: Routledge Press.

[ref41] Womack K. Zolten J. Bernhard M. (Eds) (2012). Bruce Springsteen, Cultural Studies, and the Runaway American Dream. New York City: Ashgate/Routledge.

[ref42] Yalom I. D. Leszcz M. (2020). The Theory and Practice of Group Psychotherapy. 6th Edn. New York City: Basic Books.

[ref43] Yates B. L. (2015). It’s social, not parasocial: understanding the impact of the internet on building community among Bruce Springsteen fans. Atl. J. Commun. 23, 254–268. doi: 10.1080/15456870.2015.1090438

